# Feasibility and safety of uniportal thoracoscopy for chronic pulmonary aspergillosis

**DOI:** 10.1038/s41598-023-43781-9

**Published:** 2023-09-30

**Authors:** Bing Wang, Li Yao, Jian Sheng, Xiaoyu Liu, Yuhui Jiang, Lei Shen, Feng Xu, Qibin Liu, Chao Gao, Xiyong Dai

**Affiliations:** Department of Surgery, Wuhan Pulmonary Hospital, Baofeng Road No. 28, Wuhan, Hubei China

**Keywords:** Fungal infection, Respiratory tract diseases, Outcomes research, Risk factors

## Abstract

Surgery plays a crucial role in the treatment of patients with chronic pulmonary aspergillosis (CPA). However, there is currently limited information available regarding the use of uniportal thoracoscopy (Uni-VATS) in CPA patients. To address this gap, we conducted a retrospective analysis of surgical procedures performed at a single center, aiming to demonstrate the feasibility and safety of Uni-VATS for patients with CPA. We collected basic information and surgical data from patients who underwent surgery for CPA at our hospital between January 2018 and June 2022. All patients received voriconazole antifungal medication for 3–6 months post-surgery and were monitored for a minimum of 6 months. A total of 110 patients, comprising 59 cases in the traditional open chest incision group and 51 cases in the Uni-VATS group, met the inclusion criteria. Among those who underwent surgery, 70% were male (77/110). The median age (IQR) of all enrolled patients was 55 (46–62) years. There were no statistically significant differences in general information, such as age, sex, comorbidities, BMI, FEV1, FVC, clinical symptoms, location of the disease, and duration of voriconazole antifungal medication, between the OS group and the Uni-VATS group (p > 0.05). The postoperative complication rates were 40.7% (24/59) for the traditional open chest incision group and 17.6% (9/51) for the Uni-VATS group. Through univariate analysis, we identified sex and operative approach as risk factors for postoperative complications. Multivariate logistic analysis confirmed that male and OS procedures were the independent risk factors for postoperative complications. There were statistically significant differences in operative time, intraoperative blood loss volume, postoperative drainage volume, pain scores, postoperative drainage tube removal time, postoperative hospital stay time between the OS group and the Uni-VATS group (p < 0.05). Uni-VATS is a feasible and safe surgical procedure for patients with CPA, and we recommend it as a preferred option for selected patients with CPA.

## Introduction

Chronic pulmonary aspergilloma (CPA) is a condition in which aspergillus fumigatus colonizes and grows in the lung, leading to a slow deterioration of the lung parenchyma. This can manifest as one or more cavities, nodules, infiltrates, or fibrosis, with or without the formation of an aspergilloma^1^. CPA typically occurs in patients who have a history of or are currently experiencing lung pathologies such as active or previous tuberculosis and non-tuberculosis mycobacterial infections, chronic obstructive pulmonary disease, sarcoidosis, or previous lung surgery^2^. However, the most common comorbid disease associated with CPA is tuberculosis^3^. It is important to note that CPA can be a life-threatening condition, with a mortality rate of 38%, as it can lead to severe hemoptysis resulting from the development and rupture of systemic and bronchial hypervascularization^4^.

Treatment for CPA typically involves a combination of approaches, including systemic administration of antifungal medication^5,6^, local instillation of antifungal medication^7,8^, transbronchial removal of aspergilloma^9^, embolization of the bronchial artery (BAE), and surgery. Among these options, BAE therapy is considered the most effective option for managing acute hemoptysis^10^. At present, surgery is considered the preferred treatment for symptomatic aspergilloma, although it carries the risk of trauma. According to several studies, the postoperative mortality and morbidity rates were approximately 4% and 33% respectively^11^. Minimal-invasive surgical approaches, such as video-assisted thoracic surgery (VATS), have undergone significant advancements and are now being used for more complex procedures^12^. Some researchers advocate for the use of VATS as the most appropriate method for peripheral aspergillosis without hilum infiltration, as it offers several benefits to patients, including reduced postoperative complications, decreased chest pain, faster recovery, and shorter hospital stays^13,14^. In 2010, Gonzalez-Rivas reported the first Uni-VATS lobectomy^15^, and since then, this surgical technique has gained acceptance in Asia and Europe^16–18^. Several studies have highlighted the benefits of the Uni-VATS approach, including a direct view from a sagittal plane, enhanced anatomic instrumentation, and improved exposure of the superior hilum during surgery. Furthermore, Uni-VATS reduces postoperative pain by minimizing stress to a single intercostal region and offers improved wound appearance^19–21^. In the field of lung cancer surgery, there have been no significant differences reported in terms of efficacy, complications, and prognosis between single-port and multi-port thoracoscopic approaches^22,23^. While most emergency patients with CPA and complex or massive hemoptysis in other hospitals typically undergo traditional open chest surgery (OS) or multi-port VATS, the use of Uni-VATS for patients with CPA is less common. However, in our hospital, Uni-VATS has been established as a routine minimally invasive procedure since the first case was performed in 2018. In cases where Uni-VATS cannot be performed, we immediately switch to OS surgery. Therefore, in this study, we retrospectively enrolled patients with CPA who underwent surgery over the past years to evaluate the feasibility and safety of Uni-VATS for patients with CPA.

## Materials and methods

### Ethics statement

The Wuhan Pulmonary Hospital Ethics Committee authorized this study (No. (2023)01).

### Study design

Our hospital stands as the largest center for the treatment of pulmonary disease in central China, encompassing specialized expertise in treating hemoptysis and tuberculosis. Recent data highlights the susceptibility of tuberculosis patients to aspergillus infection, giving our department a distinct advantage in managing aspergillus pneumonia. Over the years, our hospital has admitted and provided care for thousands of patients with CPA. While most patients received repeated internal medicine treatment in our department, their lung lesions persist and carry the risk of aspergillus dissemination and hemoptysis. Our specialists have identified eligible patients for surgical intervention following extensive evaluation and multi-disciplinary team discussion. This study retrospectively analyzes patients with CPA who underwent surgery using Uni-VATS and OS techniques between January 2018 and June 2022.

**Figure 1:**
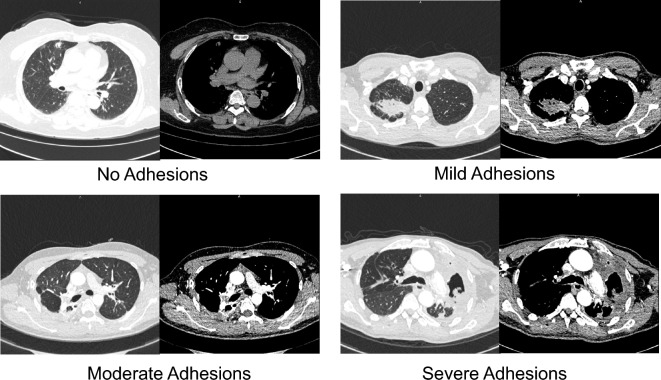
The degree of adhesions between the lung lesion and the pleura on enhanced CT.

In our study, we collected a comprehensive dataset that included various information.This information encompassed the patient's gander, age, underlying medical conditions (such as tuberculosis, diabetes, and hypertension), presence of hemoptysis, site of the disease, clinical symptoms (such as cough, sputum production, chest pain, hemoptysis, and shortness of breath), duration of voriconazole antifungal medication prior to surgery, whether the patient underwent preoperative bronchial artery embolization (BAE), body mass index (BMI), lung function measurements (FEV1 and FVC), maximum diameter of the lung lesion, degree of adhesion assessed through computer tomography (CT). Moreover, the perioperative data we collected encompassed specific surgical details. These details included the chosen method of resection (such as wedge resection, segmentectomy, or lobectomy, further categorized into sublobectomy and lobectomy groups), duration of the operation, volume of blood loss during the procedure, postoperative drainage volume within the first 24 and second 24 h, pain scores within 24 h post-operation, time for removal of the drainage tube after surgery, and length of hospital stay following the procedure. We classified clinical symptoms into two groups based on their duration of more than 6 months. Upon admission to our hospital, patients underwent prompt evaluation, and decisions regarding BAE or surgery were made based on factors like hemoptysis and vital signs. The degree of adhesion was assessed using CT imaging (Fig. 1). Nonadhesive and mild adhesions were grouped together, while moderate and severe adhesions formed another group. Additionally, we categorized the data into two groups depending on the presence or absence of short-term (30 days) postoperative complications.

### Patient selection (Fig. 2)

**Figure 2:**
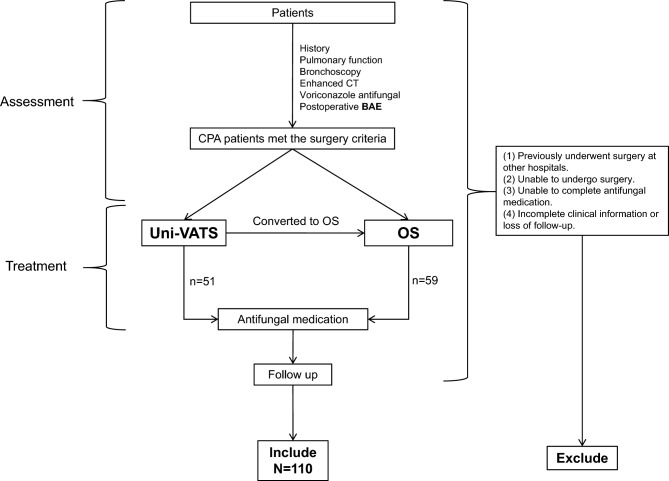
The process of treating CPA patients in our hospital.

1. Included criteria: (1) Diagnosis of CPA. (2) Underwent surgery at our hospital using Uni-VATS or traditional open chest incision. (3) Followed up for a minimum of six months after surgery , with complete clinical data available.
2. Exclusion criteria: (1) Previously underwent surgery at other hospitals. (2) Unable to undergo surgery. (3) Unable to complete antifungal medication. (4) Incomplete clinical information or loss of follow-up.

The preoperative and postoperative medication, as well as indications for surgery, were determined by multi-disciplinary team discussion which included experienced pharmacologists, imaging specialists, pulmonologists, and thoracic surgeons. Between January 2018 and June 2022, a total of 126 patients with CPA underwent surgery at our hospital. However, 12 patients were excluded from the study due to incomplete information or inconsistent surgical methods. Additionally, 4 patients were excluded due to a lack of follow-up. Ultimately, a total of 110 patients were included in this study.

### Procedures for surgery

#### Uni-VATS procedures^24–26^

This uni-VATS procedures entails making a 3–4 cm incision between the midclavicular line and anterior axillary line in the fourth or fifth intercostal space. The surgical instrument is then introduced into the chest cavity through this incision. The final step of the procedure is to place one or two chest tubes along the edge of the incision for drainage when the surgery is completed.

#### OS procedures

The incision of OS is approximately 10–20 cm. This incision passes through the intercostal or costal bed to access the thoracic cavity. A chest tube is placed in the seventh costal region along the midline axillary line, with an optional additional chest tube placed between the second or third costal region along the midline clavicle and axillary line.

### The criteria for removing the chest tube

1. Absence of effusion: This criterion is determined based on the X ray or ultrasound findings, which should show no evidence of fluid accumulation in the chest. In addition, the flow rate of the digital drainage equipment should be less than 50 ml per day, indicating minimal fluid drainage.
2. No air leak for 48 h: The chest tube must not exhibit any signs of air leakage for a continuous period of 48 h. This indicates that there is no obstructions or leaks in the chest tube system.
3. Chest image showing good lung expansion: The postoperative chest X-ray or imaging results should demonstrate satisfactory expansion of the lung. This suggests that the lung is functioning properly without any significant complications.

 

### Postoperative treatment and follow-up

All patients with CPA received a course of antifungal drug treatment with voriconazole for a duration of 3–6 months. Regular evaluations of chest CT scans were performed at 1 month, 3 months, and 6 months after surgery for all patients. If postoperative medication was administered for more than 3 months and both the chest CT scan and symptoms showed no signs of recurrence, discontinuation of antifungal medications was considered. Each patient was followed up for a period exceeding six months to monitor their condition.

### Statistical analysis

Statistical analysis was performed with SPSS software (IBM, SPSS Inc., version 24.0). Rates and percentages were used to express categorical variables which were compared with χ^2^ test or Fisher’s exact test. Continuous variables were described with Mean ± Standard Deviation (Mean ± SD) or Median (Interquartile Range) and compared with the student’s *T* test or the Mann–Whitney *U* test according to the distribution. We conducted multivariable logistic regression analysis on the variables that exhibited significant differences in the univariate analysis, and ultimately identified the corresponding risk factors. The difference was statistically significant when the p-value was less than 0.05.

### Ethics approval and consent to participate

This retrospective study was performed in accordance with the Declaration of Helsinki and approved by The Ethics Committee of the Wuhan Pulmonary Hospital (No. (2023)01). Each participant or their legal representative gave written informed consent before enrollment.

## Results

Table 1 provides an overview of the enrolled patients with CPA. Data were categorized into the OS group (59 cases) and the Uni-VATS group (51 cases) based on the surgical procedures. Among those who underwent surgery, 70% were male (77/110), with 74.6% (44/59) in the OS group and 64.7% (33/51) in the Uni-VATS group. The median age (IQR) of all enrolled patients was 55 (46–62) years, which was 55(46–62) in the OS group and 55 (46–61) in the Uni-VATS group. A total of 41.8% (46/110) of patients with CPA had a coexisting pulmonary tuberculosis infection, either currently or previously. Underlying medical conditions such as diabetes and hypertension were present in 5.5% (17/110) and 17.3% (19/110) of the patients, respectively. Among the patients with CPA, 72.7% had clinical symptoms for more than six months, and 57.3% (63/110) presented with hemoptysis. In terms of lesion location, 38.2% (42/110) were found in the right lung, while 61.8% (68/110) were in the left lung. The median duration of voriconazole antifungal medication prior to surgery was 30 (14–50) days, with similar lengths observed in the OS group (30, 14–42 days) and the Uni-VATS group (30, 21–50 days). Twelve patients underwent preoperative bronchial artery embolization for hemoptysis, with seven in the OS group and five in the Uni-VATS group. The median (IQR) BMI of all enrolled patients was 22.7 (21.8–23.7) kg/m^2^. The median (IQR) maximum diameter of lung lesions for all patients was 8 (6–10) cm. Additionally, the enhanced chest CT was used to grade the adhesion between the lung and the chest (Fig. 2). Out of the cases, 26 (23.6%) showed no or mild adhesion, 60 (54.5%) had moderate adhesion, and 24 (21.8%) exhibited severe adhesion. Among all patients, 20% underwent sublobectomy, while 80% received lobectomy. Regarding the grouping, the percentage of patients who underwent subsegmentectomy in the OS group was 11.9% (7/59), and it was 27.5% (14/51) in the Uni-VATS group. The results show that there were no statistically significant differences in general information, such as age, sex, comorbidities, BMI, FEV1, FVC, clinical symptoms, location of the disease, duration of voriconazole antifungal medication, and surgical resection method between the OS group and the Uni-VATS group (p > 0.05). Additionally, we categorized the data into two groups based on the presence or absence of postoperative complications (Table 2). Through univariate analysis, we identified gender and operative approach as risk factors for postoperative complications. Multivariate logistic analysis confirmed that male and OS procedures were the independent risk factors for postoperative complications. For more detailed information, please refer to Table 3.

**Table 1:** General information of patients enrolled.

Variable	Patients, n = 110	Operative approach (n, %)		P
		OS group, n = 59	Uni-VATS group, n = 51	
Sex (n, %)				
Male	77 (70)	44 (74.6)	33 (64.7)	0.260
Female	33 (30)	15 (25.4)	18 (35.3)	
Age (years), median (IQR)	55 (46–62)	55 (46–62)	55 (46–61)	0.950
Underlying medical conditions (n, %)				
Tuberculosis (n, %)	46 (41.8)	26 (44.1)	20 (39.2)	0.607
Diabetes (n, %)	17 (15.5)	7 (11.9)	10 (19.6)	0.263
Hypertension (n, %)	19 (17.3)	9 (15.3)	10 (19.6)	0.547
SLE (n, %)	1 (0.9)	1 (1.7)	0	1.000
Clinical symptoms > 6 months (n, %)				
Yes	80 (72.7)	43 (72.9)	37 (72.5)	0.969
No	30 (27.3)	16 (27.1)	14 (27.5)	
Hemoptysis (n, %)				
Yes	63 (57.3)	34 (57.6)	29 (56.9)	0.963
No	47 (42.7)	25 (42.4)	22 (43.1)	
Site of the disease (n, %)				
Left	42 (38.2)	26 (44.1)	16 (31.4)	0.172
Right	68 (61.8)	33 (55.9)	35 (68.6)	
Duration of voriconazole antifungal medication before surgery (days ), Median (IQR)	30 (14–50)	30 (14–42)	30 (21–50)	0.144
Preoperative BAE (n, %)				
Yes	12 (10.9)	7 (11.9)	5 (9.8)	0.730
No	98 (89.1)	52 (88.1)	46 (91.2)	
BMI (kg/m^2^), median(IQR)	22.7 (21.8–23.7)	22.8 (21.9–23.4)	22.6 (21.6–23.7)	0.642
FEV1 (L) (IQR)	2.61 (2.36–2.99)	2.48 (2.37–2.70)	2.77 (2.34–3.15)	0.600
FVC (L) (IQR)	3.61 (3.25–3.87)	3.54 (3.25–3.78)	3.67 (3.24–3.98)	0.201
Maximum diameter of lesion (cm) (IQR)	8 (6–10)	8 (6.5–10)	8 (6–10)	0.288
Degree of adhesion on CT (n, %)				
No or mild adhesion	26 (23.6)	9 (15.3)	17 (33.3)	0.060
Moderate adhesion	60 (54.5)	34 (57.6)	26 (51.0)	
Severe adhesion	24 (21.8)	16 (27.1)	8 (15.7)	
Surgical resection method (n, %)				
Subsegmentectomy	21 (20)	7 (11.9)	14 (27.5)	0.067
Lobectomy	89 (80)	52 (88.1)	37 (72.5)	

*SLE* systemic lupus erythematosus.

**Table 2:** The relationship between general information and postoperative complications.

Variable	Postoperative complications (n, %)		P
	Present, n = 33	Absent, n = 77	
Sex (n, %)			
Male	28 (84.8)	49 (63.6)	0.026*
Female	5 (15.2)	28 (36.4)	
Age (years), median (IQR)	54 (45.5–62)	56 (46–62.5)	0.829
Underlying medical conditions (n, %)			
Tuberculosis (n, %)	13 (39.4)	33 (42.9)	0.736
Diabetes (n, %)	3 (9.1)	14 (18.2)	0.375
Hypertension (n, %)	2 (6.1)	18 (23.4)	0.059
SLE (n, %)	0	1 (1.3)	1.000
Clinical symptoms > 6 months (n, %)			
Yes	22 (66.7)	58 (75.3)	0.350
No	11 (33.3)	19 (24.7)	
Hemoptysis (n, %)			
Yes	19 (57.6)	44 (57.1)	0.966
No	14 (42.4)	33 (42.9)	
Site of the disease (n, %)			
Left	13 (39.4)	29 (37.7)	0.282
Right	20 (60.6)	48 (62.3)	
Duration of voriconazole antifungal medication before surgery (days), median (IQR)	30 (14–46)	30 (21–50)	0.211
Preoperative BAE (n, %)			
Yes	4 (12.1)	8 (10.4)	1.000
No	29 (87.9)	69 (89.6)	
BMI (kg/m^2^), median (IQR)	22.8 (22.1–24.1)	22.6 (21.6–23.5)	0.202
FEV1 (L) (IQR)	2.45 (2.36–2.66)	2.65 (2.35–3.12)	0.063
FVC (L) (IQR)	3.62 (3.29–3.71)	2.45 (2.36–2.67)	0.514
Maximum diameter of lesion (cm) (IQR)	8 (6.5–10)	8 (6–10)	0.809
Degree of adhesion on CT (n, %)			
No or mild adhesion	4 (12.1)	22 (28.6)	0.118
Moderate adhesion	19 (57.6)	41 (53.2)	
Severe adhesion	10 (30.3)	14 (18.2)	
Operative approach (n, %)			
OS group*,* n = 59	24 (72.8)	35 (45.5)	0.009*
Uni-VATS group*,* n = 51	9 (27.2)	42 (54.5)	
Surgical resection method (n, %)			
Subsegmentectomy	6 (18.2)	15 (19.5)	1.000
Lobectomy	27 (81.8)	62 (80.5)	

*SLE* systemic lupus erythematosus.

*The difference was statistically significant.

**Table 3:** Results of multivariate analyses for postoperative complications.

Variables	OR	95% CI	P
Gender (male)	2.989	1.013–8.819	0.047*
Operative approach (OS)	3.037	1.230–7.4980	0.016*

*OR* odds ratio, *CI* confidence interval.

*The difference was statistically significant.

Table 4 presents the perioperative details of the surgeries. The mean duration of the operation was 280.74 ± 111.46 min, and the median blood loss (IQR) during the operation was 400 (200–800) ml. The median postoperative drainage volume (IQR) in the first 24 h was 400 (180–627.5) ml, and in the second 24 h, it was 290 (157.5–512.5) ml. The median pain scores (IQR) reported by the patients were 14 (9.75–21). The median duration of hospital stay (IQR) after surgery and the time for postoperative drainage tube removal were 14 (9.75–21) and 10.5 (7–20) respectively. However, the mean operative time of the OS group was significantly longer, with 321.90 ± 92.16 min, compared to the Uni-VATS group with 233.12 ± 113.6 min (p < 0.05). The median blood loss volume (IQR) during the operation was significantly higher in the OS group with 450 (300–1000) ml compared to the Uni-VATS group with 330 (100–500) ml (p = 0.002 < 0.05). Similarly, the median postoperative drainage volume (IQR) in the first 24 h was significantly higher in the OS group with 520 (320–820) ml compared to the Uni-VATS group with 200 (120–400) ml (p < 0.05). In the second 24 h, the OS group also had a significantly higher median postoperative drainage volume (IQR) with 420 (260–650) ml compared to the Uni-VATS group with 170 (100–300) ml (p < 0.05). Concerning the pain scores of the patients, the median (IQR) in the OS group was 9 (8–10), which was significantly higher than the Uni-VATS group with 4 (4–5) (p < 0.05). Moreover, the median (IQR) duration of hospital stay after surgery was significantly longer in the OS group with 18 (13–23) days compared to the Uni-VATS group with 12 (8–18) days (p = 0.001 < 0.05). Additionally, the median (IQR) postoperative drainage tube removal time was significantly longer in the OS group with 15 (8–21) days compared to the Uni-VATS group with 8 (5–16) days (p = 0.001 < 0.05).

**Table 4:** Perioperative details of surgery.

Perioperative details	Patients, n = 110	OS group*,* n = 59	Uni-VATS group*,* n = 51	P
Duration of the operation (minutes), Mean ± SD	280.74 ± 111.46	321.90 ± 92.16	233.12 ± 113.65	< 0.001*
Blood loss volume during operation(ml), median (IQR)	400 (200–800)	450 (300–1000)	330 (100–500)	0.002*
Postoperative dranige volume in the first 24 h (ml), median (IQR)	400 (180–627.5)	520 (320–820)	200 (120–400)	< 0.001*
Postoperative dranige volume in the second 24 h (ml), median (IQR)	290 (157.5–512.5)	420 (260–650)	170 (100–300)	< 0.001*
Pain scores, median (IQR)	5.5 (4–9)	9 (8–10)	4 (4–5)	< 0.001*
Postoperative drainage tube removal time(days), median (IQR)	10.5 (7–20)	15 (8–21)	8 (5–16)	0.001*
Hospital stay time after operation(days), median (IQR)	14 (9.75–21)	18 (13–23)	12 (8–18)	0.001*
Postoperative complication (n, %)	33 (30)	24 (40.7)	9 (17.6)	0.009*
Air leakage	21 (19.1)	14 (23.7)	7 (13.7)	0.183
Haemorrhage	6 (5.5)	4 (6.8)	2 (3.9)	0.812
Delayed wound healing	1 (0.9)	1 (1.7)	0	1.000
Chylous fistula	1 (0.9)	1 (1.7)	0	1.000
Residue cavity	1 (0.9)	1 (1.7)	0	1.000
Embolism	1 (0.9)	1(1.7)	0	1.000
Pulmoary infection (dead)	1 (0.9)	1 (1.7)	0	1.000
Bronchopleural fistula	1 (0.9)	1 (1.7)	0	1.000

*The difference was statistically significant.

### Short-term postoperative complications and management

Regarding short-term postoperative complications, the overall incidence rate was 30% (33/110). The Clavien-Dindo grading system was used to classify the complications. There were 2 patients with grade I complications, 23 with grade II, 4 with grade IIIa, 3 with grade IIIb, and 1 with grade V. Major postoperative complications included air leakage in 21 patients (2 with grade I, 15 with grade II, and 4 with grade IIIa), postoperative hemorrhage in 6 patients (4 with grade II and 2 with grade IIIb), pulmonary embolism in 1 patient (grade II), delayed wound healing in 1 patient (grade II), chylous fistula in 1 patient (grade II), residual thoracic cavity in 1 patient (grade II), bronchopleural fistula in 1 patient (grade IIIb) and lung infection in 1 patient (grade V)^27^. Out of the total cases, 24 occurred in the OS group, while 9 cases occurred in the Uni-VATS group. The incidence rate of postoperative complications in the Uni-VATS group was significantly lower than that in the OS group. In the OS group, 14 cases (23.7%) experienced lung prolonged air leakage for more than 14 days. In contrast, the percentage of patients who experienced prolonged air leakage in the Uni-VATS group was 7.3% (7/51). All patients with prolonged air leakage after surgery were successfully treated with conservative measures such as medications, lung function exercises, and adequate nutrition supplements. Four cases in the OS group experienced postoperative thoracic hemorrhage, with two of them requiring emergency surgery to control bleeding, while the other two cases were cured with hemostatic medications and blood transfusions. In the Uni-VATS group, two cases experienced postoperative thoracic hemorrhage, and both were successfully treated with conservative measures. In the OS group, one patient experienced delayed wound healing, one had chylous fistula, one patient developed a residual thoracic cavity, and one patient experienced pulmonary embolism. All of these conditions were effectively treated with conservative measures. Additionally, one patient in the OS group developed a postoperative bronchopleural fistula, which eventually resolved with thoracostomy and thoracoplasty. Another patient developed a postoperative lung infection and received ventilator-assisted treatment; unfortunately, this patient eventually passed away. At the six-month postoperative follow-up, all patients showed no evidence of local or regional recurrence (Table 2).

## Discussion

The management of patients with chronic pulmonary aspergillosis (CPA) is highly challenging and requires a multidisciplinary team of specialists, including respiratory physicians, interventionalists, and thoracic surgeons. Previous studies have found that bronchial arterial embolism can effectively prevent life-threatening hemoptysis in patients with CPA^28,29^. In our study, 12 patients (10.9%) with hemoptysis underwent preoperative bronchial arterial embolism, two of these patients experienced recurrent hemoptysis before lung resection surgery. Neither medications nor bronchial artery interventions can completely cure lung cavities in CPA patients or prevent massive hemoptysis caused by vessel hyperplasia in the chest wall or para bronchus. Therefore, surgical intervention is often necessary to block proliferating trophoblastic vessels and remove the damaged lung tissue in CPA patients. In these cases, dense adhesions typically form between the affected lung lobes and the pleura, along with a significant number of new trophoblastic vessels within the adhesions. It is crucial to separate these adhesions and address the trophoblastic vessels during the operation. Recent studies have demonstrated that minimally invasive thoracoscopic surgery, when compared to open surgery, provides several advantages. These include reduced pain, fewer complications, smaller incisions and shorter hospital stays. Minimally invasive thoracoscopic surgery typically results in less postoperative pain compared to traditional open surgery. This can lead to improved patient comfort and a more rapid recovery. Minimally invasive techniques have been associated with a lower rate of complications when compared to open surgery. This may include a reduced risk of infection, bleeding, and other surgical complications. Thoracoscopic surgery utilizes small incisions, often only a few centimeters in length, as opposed to larger incisions required for open surgery. Smaller incisions can result in less scarring, decreased tissue trauma, and improved cosmetic outcomes. Minimally invasive procedures generally require a shorter hospital stay compared to open surgery. This can lead to faster overall recovery and a quicker return to daily activities. These advantages make minimally invasive thoracoscopic surgery an attractive option for many patients, offering the potential for improved outcomes and a better overall surgical experience.^30,31^. The effectiveness of thoracoscopy in CPA patients has also been supported by various studies^13,32^. Uni-VATS is increasingly utilized in thoracic diseases^33,34^, but its use in CPA patients is limited. Uni-VATS has been shown to alleviate postoperative pain and aid in recovery by confining surgical trauma to a single intercostal space^35,36^. OS requires incisions on the patient’s chest wall, typically ranging from 10 to 20 cm in size. In contrast, Uni-VATS thoracoscopic surgery only requires a single incision, usually about 3 cm in size. The incision for Uni-VATS is typically made in the axilla, anterior chest, or lateral chest, allowing for the insertion of a camera and surgical instruments at different angles to perform the operation. The small incision in Uni-VATS helps reduce the risk of tissue damage and bleeding, and it contributes to a shorter recovery time post-operation. Additionally, the unique small incision can improve cosmetic outcomes by minimizing scar formation. Moreover, Uni-VATS provides better visualization of the pleural apex area compared to OS, where the view is narrower between the upper lung and the pleural apex.

Based on the results of our study, we observed no no significant differences in general patient characteristics. Additonally, there was no difference in preoperative embolization between the two groups. In terms of perioperative surgical details, there were no statistically significant variances in the surgical resection method performed between the two groups. The extent of lung lesions and the degree of adhesions to the chest wall were also similar in both groups. The overall postoperative complication rate in patients with CPA was found to be 30%, which aligns with the rates reported in other studies^37^. Interestingly, our study indicated an incidence of surgical complications of approximately 40% in the OS group and 17% in the Uni-VATS group, suggested a significant difference between the two groups. Our finding demonstrated that OS was a risk factor for the development of postoperative complications, potentially due to the gentle operation, meticulous hemostasis, and reduced trauma of Uni-VATS^30^.

In terms of duration of the operation , we observed that the Uni-VATS group had a shorter duration compared to the OS group, which contradicts existing literature on Uni-VATS in other diseases such as lung cancer. There are several reasons that could explain this result. Firstly, our routine practice involves performing thoracoscopic exploration before deciding on a single-port thoracoscopic procedure, based on intraoperative findings. Secondly, Uni-VATS procedures have advantages in terms of chest opening and closure. Thirdly, Uni-VATS allows for improved access to blind spots encountered in open surgery and has a distinct advantage in managing thoracic adhesions and thoracic blood leakage, especially in locations like the pleural apex. As a result, the total operative time for Uni-VATS was shorter compared to OS. Additionally, the volume of blood loss during the procedure was lower in the Uni-VATS group compared to the OS group. In our study, we separately assessed postoperative drainage volume in the first 24 h and the second 24 h, revealing that the OS group had higher drainage volume compared to the Uni-VATS group. The reduced intraoperative bleeding and postoperative drainage in the Uni-VATS group provide substantial evidence of its less-invasive nature for patients with CPA. Previous research by Yang et al. demonstrated that Uni-VATS was associated with less postoperative discomfort, significantly lower paresthesia rates, and shorter hospital stays in their systematic review and meta-analysis^38^. Larger incisions in OS result in more damage to the ribs, muscles, and thoracic nerves, leading to relatively higher postoperative pain scores. In our study, patients in the Uni-VATS group had significantly lower pain scores in the 24-h postoperative period compared to those in the OS group, aligning with the existing literature on the effectiveness of Uni-VATS in reducing postoperative pain scores in thoracic surgery^39^. The removal time of postoperative drainage tubes mainly depends on the recovery of chest and lung trauma, and our study revealed that the time for chest tube removal after Uni-VATS was shorter than in the OS group, consistent with the current literature^40^. Furthermore, our study demonstrated that the postoperative hospital stay was significantly shorter for patients in the Uni-VATS group compared to the OS group, consistent with findings in the current literature^35^. Overall, our study highlights that Uni-VATS significantly reduces the duration of operation, postoperative pain levels, blood loss during the procedure, duration of chest tube placement, length of hospital stay, and the overall rate of complications. These results further support the feasibility of Uni-VATS in select patients with CPA.

However, it is important to acknowledge the limitations of our research. Firstly, this study is a retrospective analysis conducted in a single center, lacking the benefits of a prospective cohort design. This may limit the robustness of the findings and the ability to draw definitive conclusions. Secondly, the sample size in our study was relatively small, potentially impacting the generalizability of the results. A larger sample size would be desirable for more representative findings.Thirdly, we were unable to include certain laboratory data, such as hemoglobin, white blood cell count, and procalcitonin levels before and after surgery, which could have provided valuable insights into the patients’ condition and response to the procedure. These limitations highlight the need for further refinement and improvement in future studies. Prospective studies involving multiple centers with larger sample sizes and more comprehensive data collection would help strengthen the evidence base for the application of Uni-VATS in patients with CPA.

In conclusion, based on our findings, it appears that the application of Uni-VATS in patients with CPA does not increase the risk of surgical complications or mortality. This suggests that Uni-VATS is a feasible and safe surgical approach for selected patients with CPA. Uni-VATS should be considered as a preferred procedure, but within the context of individual patient characteristics, surgeon expertise, and available resources.

## Data Availability

All data are available from the corresponding author by email request.
